# The kinetochore-dependent and -independent formation of the CDC20-MAD2 complex and its functions in HeLa cells

**DOI:** 10.1038/srep41072

**Published:** 2017-01-23

**Authors:** Jianquan Li, Nanmao Dang, Daniel James Wood, Jun-Yong Huang

**Affiliations:** 1 Institute for Cell and Molecular Biosciences, Newcastle University, Framlington Place, Newcastle upon Tyne, NE2 4HH, UK

## Abstract

The mitotic checkpoint complex (MCC) is formed from two sub-complexes of CDC20-MAD2 and BUBR1-BUB3, and current models suggest that it is generated exclusively by the kinetochores after nuclear envelope breakdown (NEBD). However, neither sub-complex has been visualised *in vivo*, and when and where they are formed during the cell cycle and their response to different SAC conditions remains elusive. Using single cell analysis in HeLa cells, we show that the CDC20-MAD2 complex is cell cycle regulated with a “Bell” shaped profile and peaks at prometaphase. Its formation begins in early prophase before NEBD when the SAC has not been activated. The complex prevents the premature degradation of cyclin B1. Tpr, a component of the NPCs (nuclear pore complexes), facilitates the formation of this prophase form of the CDC20-MAD2 complex but is inactive later in mitosis. Thus, we demonstrate that the CDC20-MAD2 complex could also be formed independently of the SAC. Moreover, in prolonged arrest caused by nocodazole treatment, the overall levels of the CDC20-MAD2 complex are gradually, but significantly, reduced and this is associated with lower levels of cyclin B1, which brings a new insight into the mechanism of mitotic “slippage” of the arrested cells.

The spindle assembly checkpoint (SAC) monitors the faithful segregation of the sister chromatids in mitosis by detecting kinetochore attachment^1^,^2^. It is believed that there is a diffusible “anaphase wait” signal which is initiated by unattached kinetochores and that even a single kinetochore will generate a sufficiently large signal to delay the metaphase/anaphase transition^3^,^4^. This signal functions by inhibiting the anaphase promoting complex or cyclosome (APC/C), an E3 ubiquitin ligase, which marks key mitotic regulators, such as cyclin B1 (CCNB1) and securin, for destruction and so prevents the premature onset of anaphase^1^,^5^.

It was previously thought that the SAC signal was controlled in an “on” and “off” manner, and that it was activated after nuclear envelope breakdown (NEBD) in late prophase or early prometaphase in response to unattached kinetochores, and was then rapidly turned off at the metaphase/anaphase transition^1^,^2^. The MCC (mitotic checkpoint complex), containing the four proteins BUBR1, BUB3, CDC20 and MAD2, has been widely referred to as the predominant inhibitor of the APC/C at the molecular level, and is probably formed from two sub-complexes, CDC20-MAD2 and BUB3-BUBR1^1^,^2^. This kinetochore-dependant “on” and “off” mechanism has been challenged by the suggestion that the SAC signal is controlled like a “rheostat switch”, where its strength depends on the amount of MAD2 recruited to the kinetochores and on the amount of MCC formed which are determined indirectly either by depletion of MAD2 or inhibition of MPS1^6^. However, despite the MCC playing a central role in SAC function, exactly how the MCC assembly is regulated in terms of the strength of the SAC in normal mitotic progression has yet to be revealed.

The primary target of the SAC is CDC20/Fizzy, which it incorporates into the MCC so blocking the APC/C function as CDC20 is an essential activator of the APC/C^7^. Ultimately, the SAC needs to respond to unattached kinetochores. The popular “MAD2 template” model suggests that recruitment of MAD2 by unattached kinetochores plays a crucial role in promoting the formation of the CDC20-MAD2 complex^8^,^9^ and that subsequently this complex combines with the BUB3-BUBR1 sub-complex to form the MCC^1^. In contrast to the kinetochore-dependent formation, it has been suggested that the MCC can be isolated from interphase HeLa cell extract, and that SAC inhibition of the APC/C was due to the selective activation of the MCC, rather than changes in the amounts of complex being generated. This, however, remains to be tested^10^,^11^. In *S. cerevisiae*, the Cdc20-Mad2 sub-complex, or the MCC, is still detectable in checkpoint-defective cells in which Ndc10, an essential protein for core kinetochore assembly, has been mutated^12^,^13^. This, together with many other observations^14^,^15^,^16^, suggests that the SAC signalling network might operate independently of the kinetochores. Therefore, examining individual cells to investigate when the MCC forms during the cell cycle and in which cellular compartment will provide clues as to whether the process is or is not kinetochore-dependent.

In the presence of an active SAC, most cells ultimately escape mitosis and enter the next G1 as tetraploid cells^17^. This process is referred to as mitotic slippage^18^,^19^ and occurs because the SAC cannot completely inhibit the APC/C to prevent a slow but continuous degradation of cyclin B, which eventually allows the cell to escape from mitotic arrest^19^. The slippage results in aberrant chromosome segregation, and the failure of cytokinesis, which can drive polyploidy, chromosome instability, and cancer formation^20^. However, the mechanisms that produce a persistent low activity of the APC/C in this scenario remain elusive.

In attempting to address these problems, we have examined the temporal and spatial profile of the *in vivo* protein-protein interaction between CDC20 and MAD2 from fixed individual HeLa cells, using an Duolink based *in situ* proximity ligation assay (PLA)^21^ combined with a functional analysis using conventional biochemical approaches. PLA is one of a few widely used^22^,^23^,^24^,^25^ and commercially available methods for analyzing protein-protein interactions in their native state (http://www.olink.com/products/duolink/applications/protein-interactions). Duolink PLA technology makes use of two primary antibodies raised in different animal species to target two proteins of interest in fixed individual single cells. Species-specific secondary antibodies (PLA probes), each conjugated with a unique short oligonucleotide tail, bind to the primary antibodies. When the PLA probes are in close proximity (<40 nm), these oligonucleotide tails can then act as a template for rolling circle amplification, and each of this amplification will produce a dot of fluorescent signal when labelled by complementary oligonucleotide probes. This amplified fluorescent signal can be detected and quantified based on microscopy images^21^,^26^,^27^, for instance a laser scanning confocal system with appropriate excitation wavelengths. Therefore, the PLA can avoid biochemical extraction or the creation of exogenous over-expressed fusion proteins and can assign signals to specific subcellular locations. For the first time, we have shown the profile of the CDC20 and MAD2 interaction detected in single cells throughout the cell cycle. Together with functional analysis we have provided clear evidence, which helps to clarify some important concepts regarding the SAC mechanism.

## Results

### The amount of CDC20-MAD2 interaction increased and decreased in a “bell” shaped manner with the peak at prometaphase

**Figure 1:**
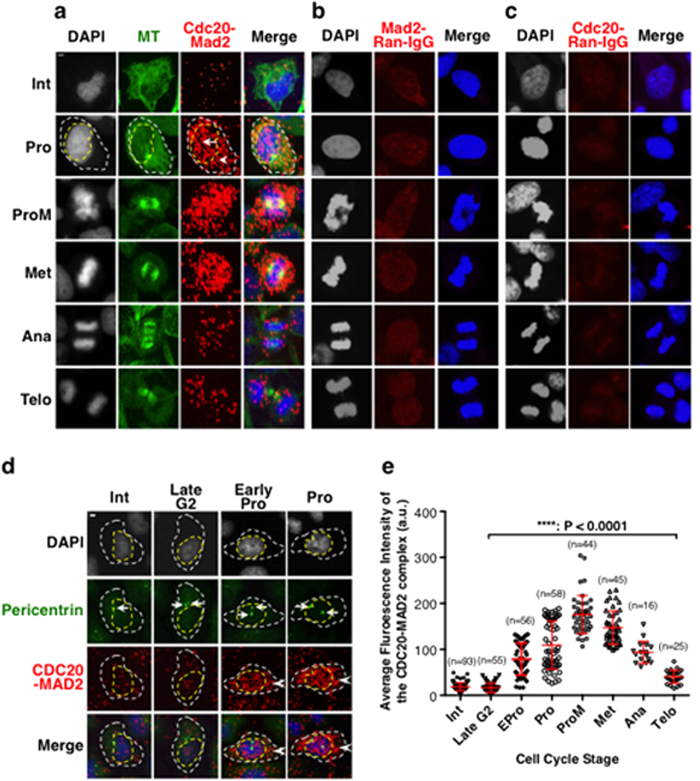
The interaction between CDC20 and MAD2 is cell cycle regulated with the levels high during mitosis, but it is also detectable during anaphase in unperturbed HeLa cells. Projected Z-stack sectional confocal images showing examples of the PLA signals between CDC20 and MAD2 (**a**), but not between pairs of MAD2-Random IgG (**b**) and CDC20-Random IgG (**c**) at selected cell cycle stages. White and yellow dash circle lines highlight the cytoplasmic region and the nuclear region of a prophase cell respectively. Arrows and arrowheads highlight the fluorescent signals detected in the nucleus and cytoplasm respectively. DNA (in blue) and microtubule (in green) morphologies were used to determine the cell cycle stages. (**d**) HeLa cells were stained with pericentrin antibody to mark the centrosomes (in green). DNA (blue) and centrosome morphologies were used to determine the cell cycle stages and this allows the separation of the cell cycle stages before prophase into interphase, late G2 and early prophase as indicated. The white dash circle lines highlight the cell boundaries and the arrows highlight the centrosomes. (**e**) Showing the quantitative profile of the interaction between CDC20 and MAD2 illustrated by the average PLA fluorescence intensities from entire cellular regions (white encircled dash lines indicated in a & b) of individual cells at different cell cycle stages. n: The number of cells used for quantification. ****P < 0.0001. Standard deviation bars are in red. The images were produced using a Leica TCS SP2 confocal system using sequential + Z-stack scanning mode with consistent system settings. Int: Interphase, L-G2: Late G2, Pro: Prophase, ProM: Prometaphase, Met: Metaphase, Ana: Anaphase, Telo: Telophase. Scale bar = 5 μM.

It is widely accepted that the inhibitory “on” signal of the SAC is generated exclusively by unattached kinetochores^1^,^2^, and that the MCC, formed between the two sub-complexes of CDC20-MAD2 and BUBR1-BUB3, is the most potent “on” signal. However, this contradicts various biochemical studies which have suggested that the MCC or CDC20-MAD2 complex exists in interphase cells^10^,^11^,^28^,^29^. Analysis of the *in vivo* formation of the CDC20-MAD2 complex using unperturbed single cells allows us to clarify this discrepancy. The Duolink based PLA can detect protein-protein interactions in their native state based on single cell analysis and has been widely used for *in vivo* studies^21^,^22^,^23^,^24^,^25^, though not of the mitotic checkpoint components. To examine the temporal and spatial profile of the interaction between CDC20-MAD2 from fixed individual HeLa cells, we therefore first tested the use of PLA in detecting the CDC20-MAD2 complex by pairing antibodies of mouse monoclonal anti-CDC20 antibody (Santa Cruz, p55CDC (E-7, sc-13162) with rabbit anti-MAD2 (Bethyl, A310-082A, which has been recommended for PLA use); and rabbit polyclonal anti-CDC20 antibody (Abcam, ab26483) with mouse monoclonal anti-MAD2 antibody (Santa Cruz, sc-47747). The various cell cycle stages were determined using microtubule and DNA morphologies. The results show both pairs produced similar profiles for the interaction between CDC20 and MAD2 throughout the cell cycle after images were projected from Z-stack confocal scans to reveal the total fluorescent signal (Fig. 1a and S1). The results show that the level of the fluorescent signals representing the potential interactions between CDC20 and MAD2 are cell cycle regulated and seems to be very low in interphase cells. Hereafter, unless otherwise stated, the PLA signals representing the interaction between CDC20 and MAD2 were produced using mouse monoclonal anti-CDC20 antibody (Santa Cruz, p55CDC (E-7, sc-13162) and rabbit anti-MAD2 (Bethyl, A310-082A).

**Figure 2:**
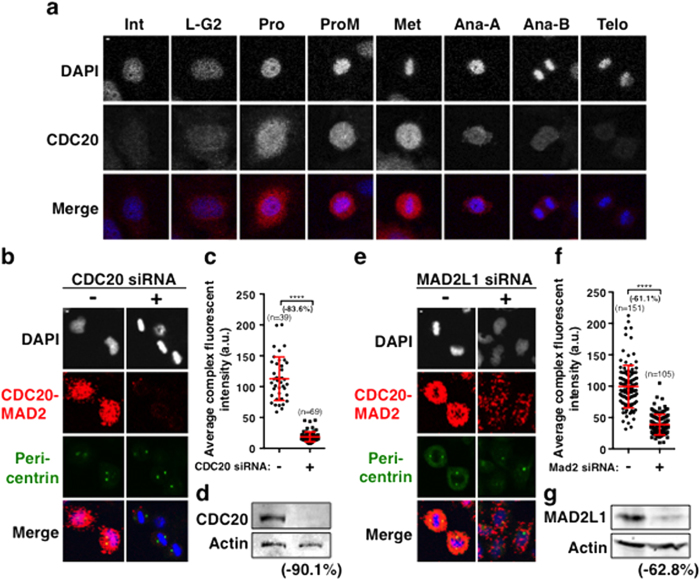
Showing the CDC20 protein expression profile throughout the cell cycle and the specificities of the antibodies. (**a**) Confocal time-lapse images showing the expression profile of CDC20 (grey in middle panel and red in merged panel) throughout the cell cycle stages as indicated. DNA was stained by DAPI (grey in top panel and blue in merged panel). Int: interphase; L-G2: late G2; Pro: prophase; ProM: prometaphase; Met: metaphase; Ana-A: anaphase A; Ana-B: anaphase B, and Telo: telophase. CDC20 was stained with a rabbit polyclonal anti-CDC20 antibody (Santa Cruz, sc-8358) and a rabbit anti-Cy3 secondary antibody both in 1:500 dilutions. Scale bar = 5 μM. (**b**–**d**) Showing the CDC20-MAD2 PLA signals in cells with or without the depletion of CDC20 using siRNA for 48 hours. Representative images were show in (**b**). The quantitative results were shown in (**c**). Western blot results indicated the reduction of the endogenous protein of CDC20 in (D) with the same sample order as shown in (**b**) or (**c**). (**e**–**g**) Showing the CDC20-MAD2 PLA signals in cells with or without the depletion of MAD2L1 using siRNA for 48 hours. Representative confocal images were show in (**e**). The quantitative results were shown in (**f**). Western blot results indicated the reduction of the endogenous protein of MAD2L1 in (**g**) with the same sample order as shown in (**e**) or (**f**). DNA (DAPI stain) in grey and blue in merged images. Centrosomes (staining with a pericentrin antibody combined with a FITC secondary antibody) in green. n: The number of cells used for quantification. The fluorescent signals of the CDC20-MAD2 interaction were visualised by TexasRed in red. Scale bar = 5 μM. P value, ****p < 0.0001.

As it is difficult to distinguish between interphase, late G2 and early prophase cells based on the morphologies of microtubules and DNA, we have analysed the CDC20-MAD2 complexes at these specific stages in more detail, determining the specific stages by comparing the position of the separated centrosomes using anti-pericentrin antibody staining and DNA morphology (DAPI staining) (Fig. 1d). The quantification of the average fluorescence intensity of the interaction between CDC20 and MAD2 throughout the cell cycle is then displayed (Fig. 1e) and the results confirm that the level of the CDC20-MAD2 complexes remains at a basal level in interphase, starts to increase after late G2 and early prophase, it continues to increase before NEBD in late prophase and early prometaphase, and peaks at prometaphase, and then gradually declines to a basal level in telophase (Fig. 1e). Substantial amounts of the complex are still present in anaphase cells when the SAC is supposed to be turned off. This probably should not be too surprising since the MAD2 level remains constant throughout the cell cycle and CDC20 is only degraded by the APC/C^Cdh1^ in late mitosis and G1^30^,^31^,^32^ with substantial amounts of CDC20 still detectable in anaphase B HeLa cells (Fig. 2a). Thus the interaction between CDC20 and MAD2 occurs with a “bell” shaped profile rather than in an “on” and “off” manner, and the existence of the complex in both prophase and anaphase suggests that there is a threshold mechanism which controls the SAC function.

In order to test the specificity of the CDC20 and MAD2 antibodies used for detecting the CDC20-MAD2 interaction, the primary antibody against either CDC20 or MAD2 was paired with an appropriate normal non-specific rabbit or mouse IgG (called a random IgG hereafter). The results (Fig. 1b,c) showed that no fluorescent signals were detected from interactions between CDC20-random IgG and MAD2-random IgG. We then tested this by depleting the CDC20 and MAD2 from culture cells using siRNA prior to PLA staining. The results showed that it achieved about a 90% reduction in the level of endogenous CDC20 and 63% in the level of MAD2 protein respectively (Fig. 2d and g). After depletion there were about 84% and 61% reductions in the CDC20-MAD2 complex fluorescent signals when PLA staining was performed as described above (Fig. 2b,c and e,f). Thus, the fluorescent signals detected by the PLA are specific to the interactions between CDC20 and MAD2, despite the potential for non-specific signals to be produced if there are two particles, which are not interacting but are less than 40 nm apart^21^.

**Figure 3:**
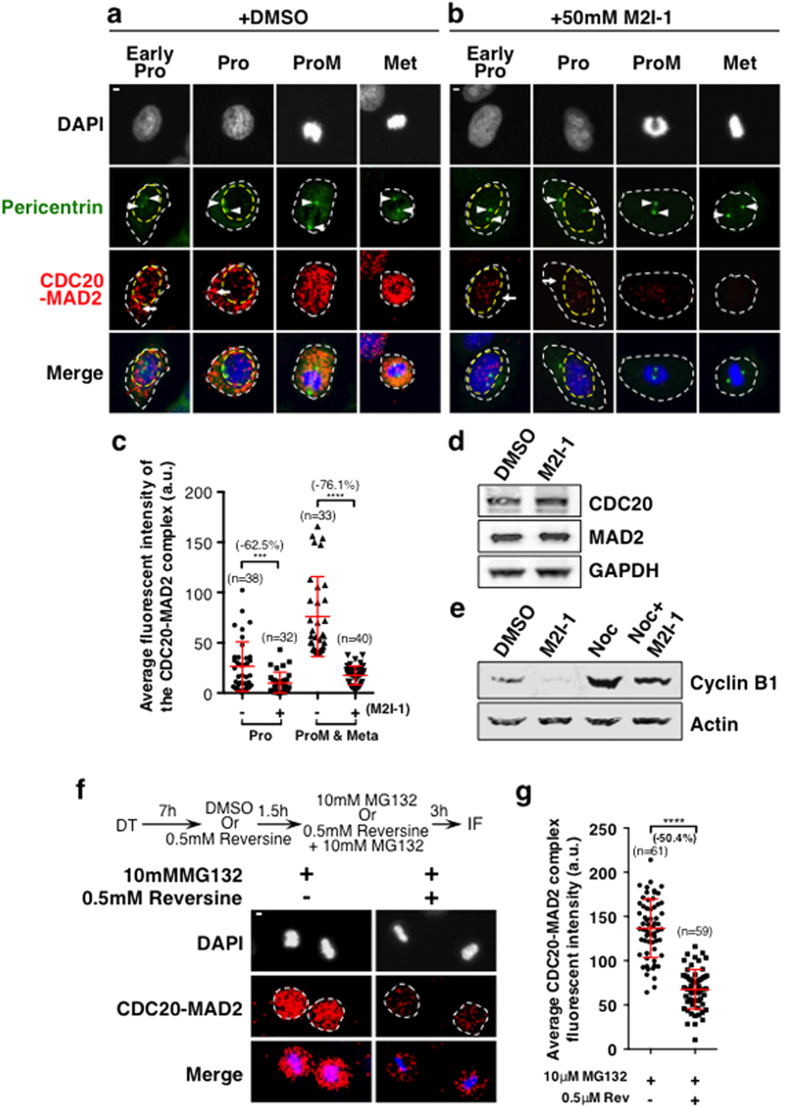
The PLA fluorescent signals detected using the antibodies to CDC20 and MAD2 reflect the physical interaction between these two proteins *in vivo*. HeLa cells were treated with 0.5% DMSO (control in **a**) or 50 μM M2I-1 in 0.5% DMSO (**b**) for 16 hours. The PLA signals (TexasRed) of the CDC20-MAD2 were quantified from projected confocal images of relevant Z-scan stacks from cells at different cell cycle stages as indicated. Arrows indicate the cytoplasmic regions of the prophase cells. DNA morphologies (DAPI staining in grey in the top panel and blue in the bottom panel) and centrosome morphologies (Arrowheads, pericentrin staining in green) were used to determine the cell cycle stages. The white dash circle lines are used to highlight the boundaries of the cell or nucleus where appropriate. (**c**) Quantitative results comparing the PLA signals between DMSO (control) and 50 μM M2I-1 in 0.5% DMSO treated cells. A two-tailed unpaired t-test was used to assess the significance between the two groups as indicated. Statistical significance was assigned where ***P < 0.0008, ****P < 0.0001. Standard deviation bars are in red. (**d**) Western blot results showing that similar levels of CDC20 and MAD2 are found in the samples extracted from the cells with or without M2I-1 treatment. GAPDH is the loading control. (**e**) Western blot results showing cyclin B1 levels in the relevant samples. Actin is the loading control. (**f**) HeLa cells released after 7 hours of thymidine synchronization, were then treated with either 0.1% DMSO as the control or 0.5 μM reversine in 0.1% DMSO for 1.5 hours and then for another 3 hours with either 10 μM MG132 in 0.1% DMSO or 10 μM MG132 and 0.5 μM reversine in 0.1% DMSO. Cells were then fixed and PLA treated as described before. (**g**) The quantitative average PLA fluorescent intensities from individual arrested prometaphase and metaphase cells encircled with the dash lines in (**f**) as examples of the regions of interest. P value: ****p < 0.0001. n: The number of cells used for quantification. Scale bar = 5 μM.

To test if these PLA signals reflect genuine interactions between CDC20 and MAD2, PLA was performed after HeLa cells were treated for 16 hours with 50 μM M2I-1 (MAD2 inhibitor-1) (Fig. 3a and b). M2I-1 is a small molecule that has been shown to disrupt complex formation between CDC20 and MAD2^33^. We anticipated that if the fluorescent signals (Fig. 1a and d) reflected a genuine interaction between the two proteins it would disappear or be significantly reduced after treatment with M2I-1. Representative images at different stages of the cell cycle (Fig. 3a,b) and the quantitative results (Fig. 3c) both reveal the reductions of the CDC20-MAD2 PLA signals after M2I-1 treatment. Quantitatively, there was about a 62.5% (Fig. 3c) reduction in cytoplasmic regions of treated cells during prophase as indicated with white arrows (Fig. 3a and b) and collectively 76.1% (Fig. 3c) in cells that possessed prometaphase and metaphase morphologies from the populations examined. The PLA signals were quantified from across the entire cell regions encircled by the white dash lines as indicated (Fig. 3a and b). These reductions are comparable to the *in vitro* biochemical analysis where an 86% reduction has been observed using recombinant proteins treated with the same concentration of M2I-1^33^. The reduction of these signals is not due to the loss of either of the two proteins as the western blot results confirmed that similar concentrations of CDC20 and MAD2 are found in the treated cells compared to the control (Fig. 3d), and in agreement with the anticipated consequence of disrupting the interaction between CDC20 and MAD2, the western blot results also show an obvious reduction in the cyclin B1 levels in samples treated with M2I-1 alone or M2I-1 combined with nocodazole (Fig. 3e).

MPS1 is also a component of the SAC and its kinase activity is known to be required for MCC formation and reversine is a potent mitotic inhibitor of MPS1^34^,^35^,^36^. To further validate the specificity of the PLA signal representing the interaction between CDC20-MAD2, PLA was performed after HeLa cells were treated with 0.5 μM reversine together with 10 μM MG132 or MG132 alone in DMSO as the control^35^,^36^. The addition of the MG132 is to prevent the degradation of the CDC20 and slippage, as the inhibition of MPS1 would sharply accelerate anaphase onset^34^,^36^. Results suggest that there was about 50% reduction in the CDC20-MAD2 PLA signals (Fig. 3f and g). This is in consistent with the previous observation, using co-immunoprecipitation, of an approximately 50% reduction in MAD2 incorporation into the MCC after similar treatment in HeLa cells^36^. Thus the fluorescent signals displayed in Fig. 1a and d reflect the specific interaction between CDC20 and MAD2 during the cell cycle.

### Tpr can facilitate the formation of the CDC20-MAD2 complex in prophase cells independent of the SAC

**Figure 4:**
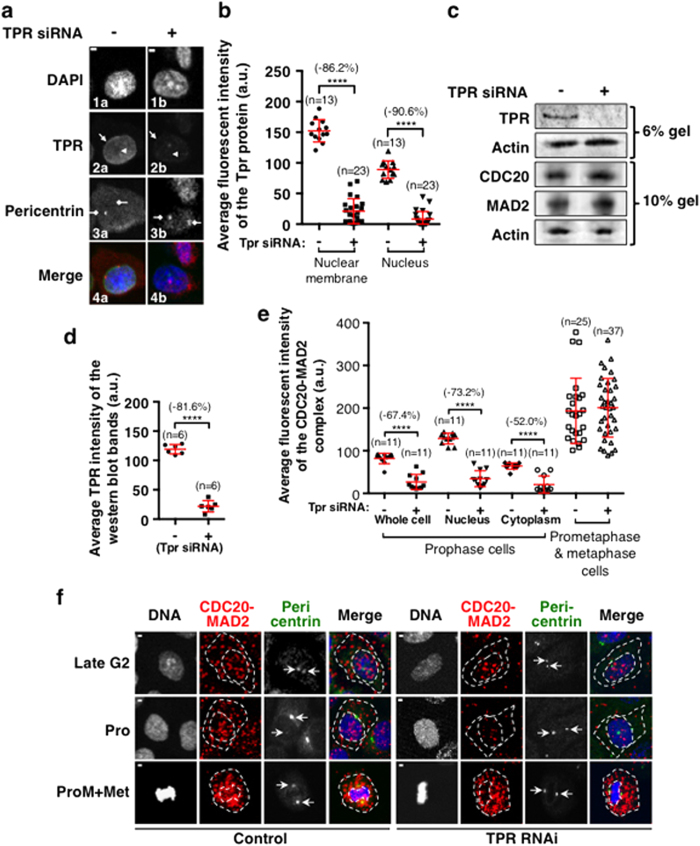
Tpr can facilitate the formation of the CDC20-MAD2 complex in prophase cells independent of the SAC. (**a**) Confocal images showing the Tpr protein levels in prophase cells, control: left column, grey in 2a and green in 4a; and Tpr siRNA treated: right column, grey in 2b and green in 4b. DNA was stained with DAPI and is shown in grey (1a,b) and blue (4a,b). Tpr was stained with a rabbit primary antibody to Tpr (sc-67116, Santa Cruz). Centrosomes were stained by a mouse monoclonal primary antibody to pericentrin (ab28144) and are show in grey (3a and b) and in red in the merged images (4a and b). The solid white arrows highlight the nuclear membranes and the white arrows with dashed line highlight the nucleus. The diamond-head white arrows highlight the centrosomes. (**b**) Quantitative comparison of the Tpr protein fluorescent levels on the nuclear membranes and in the nucleus between the control and Tpr siRNA cells in prophase as exampled in A. (**c**) Western blot results showing the reduction of the Tpr protein level in Tpr siRNA cells compared to the control. Actin acts as the loading control. (**d**) Quantitative comparison of the Tpr protein levels from western blot, data from 4 independent western blot results. (**e**) The quantitative results showing the average fluorescent intensities quantified from cytoplasmic and nuclear compartments of individual cells at cell cycle stages as indicated between control and Tpr siRNA cells. (**f**) Example confocal images showing the CDC20-MAD2 complex profiles detected in control and Tpr siRNA cells at cell cycle stages as indicated. DNA was stained with DAPI and is shown as described above. Centrosomes (white arrows) were stained with a rabbit primary anti-human pericentrin 1 & 2 (ab4448) in grey or in green in the merged images. The PLA detected CDC20-MAD2 complex fluorescent signals are shown in red. The cellular boundaries were defined by the non-specific staining of the pericentrin signals and the nuclear boundaries were defined by the DAPI staining, both of them are highlighted by the white dashed lines. n: The number of cells used for quantification. Scale Bar = 5 μM. ****P < 0.0001.

Our findings suggest that there is only a basal level of CDC20-MAD2 complex in interphase cells and that it starts to accumulate in prophase cells before NEBD when the SAC, it is generally believed, has not been activated by the unattached kinetochores^1^,^2^. This once again suggests that there are alternative ways to trigger the formation of this complex which are not kinetochore dependent. More recently, biochemical approaches, have suggested that Tpr/Megator (in flies)/MPLs (myosin-like proteins in budding yeast), one of the components of the nuclear pore complexes (NPCs) can trigger the APC/C-inhibitory signaling mediated by the MAD1-MAD2 complex in “interphase” cells^37^,^38^,^39^,^40^ but not in prometaphase and metaphase cells^41^. Therefore, Tpr might be involved in signaling the formation of this prophase form of the CDC20-MAD2 complex independently of the SAC. Hereafter, this complex will be referred to as the PCM for “Prophase CDC20-MAD2” to separate from the MCC sub-complex of CDC20-MAD2 in mitosis. Previous data have shown that Tpr interacts with MAD1 and MAD2 throughout the cell cycle and is required to maintain the stability of these proteins^40^, but this contradicts the findings that the depletion of Tpr did not affect the amount of the interaction of CDC20 and MAD2 from mitotic extracts^41^. To test if the formation of the PCM complex is specifically Tpr dependent and if its levels in prometaphase and metaphase are also reduced after Tpr depletion because of the destabilization of the MAD2 protein, siRNA treatments were conducted to transiently knockdown Tpr in HeLa cells. The results showed that after 96 hours of Tpr siRNA treatment, there were significant reductions in the levels of the Tpr protein (Fig. 4a) both on the nuclear membrane (86.2%) and in the nucleus (90.6%) (Fig. 4b). An overall 82% reduction of the Tpr protein level is also confirmed by quantification of western blot results (Fig. 4c,d). With the Tpr depletion, the PCM complex from nuclear and cytoplasmic compartments of prophase cells was reduced by about 73.2% and 52.0% respectively, giving an overall reduction of 67.4% (Fig. 4e,f). In contrast, the results show that at prometaphase and metaphase there is no significant difference between the CDC20-MAD2 complex levels in Tpr depleted and control cells (Fig. 4e,f). Western blot results show that there is no detectable change in MAD2 protein levels between the samples after Tpr depletion and control cells (Fig. 4c). This, together with the observations of previous studies, suggests that Tpr can facilitate the formation of the PCM complex in prophase cells independent of the SAC, but does not affect the formation of this complex in prometapase and metaphase.

### The PCM complex is functional, allowing the accumulation of cyclin B1 independent of the SAC

**Figure 5:**
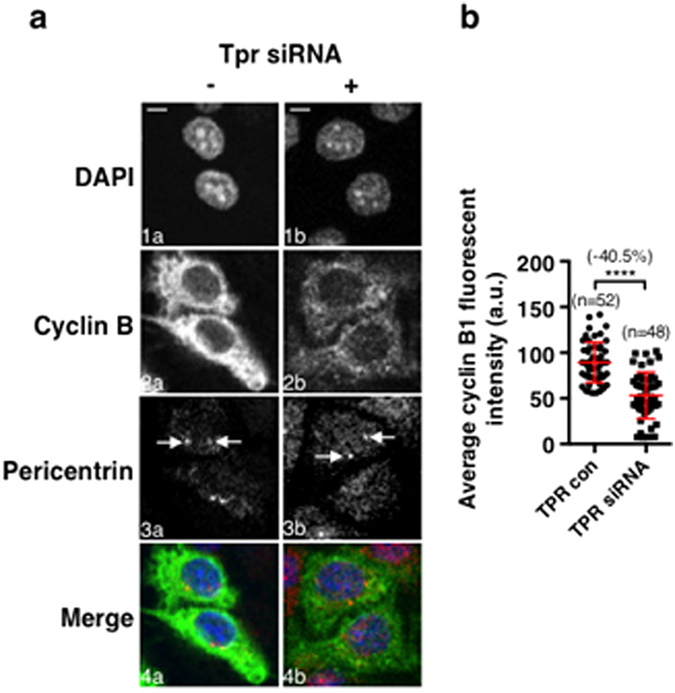
The specific form of the CDC20-MAD2 complex found in prophase cells is functional allowing the accumulation of cyclin B1 independent of the SAC. (**a**) An example of the confocal images showing cyclin B1 profiles between control and Tpr siRNA cells in prophase. Cyclin B1 was stained using the rabbit polyclonal anti-cyclin B1 (H-433) (sc-752) antibody and is shown in grey (2a and b) or in green in the merged images (4a and b). Centrosomes (white arrows) were stained using a mouse monoclonal primary anti-human pericentrin (Abcam ab28144) and are shown in grey (3a and b) or in red in the merged images (4a and b). DNA was stained with DAPI and is shown in grey (1a and b) or in blue in the merged images (4a and b). (**b**) Quantitative results showing the cyclin B1 levels in individual prophase control and Tpr siRNA treated cells for comparison. ****P < 0.0001. Standard deviation bars are in red. n: The number of cells used for quantification. Scale Bar = 5 μM.

**Figure 6:**
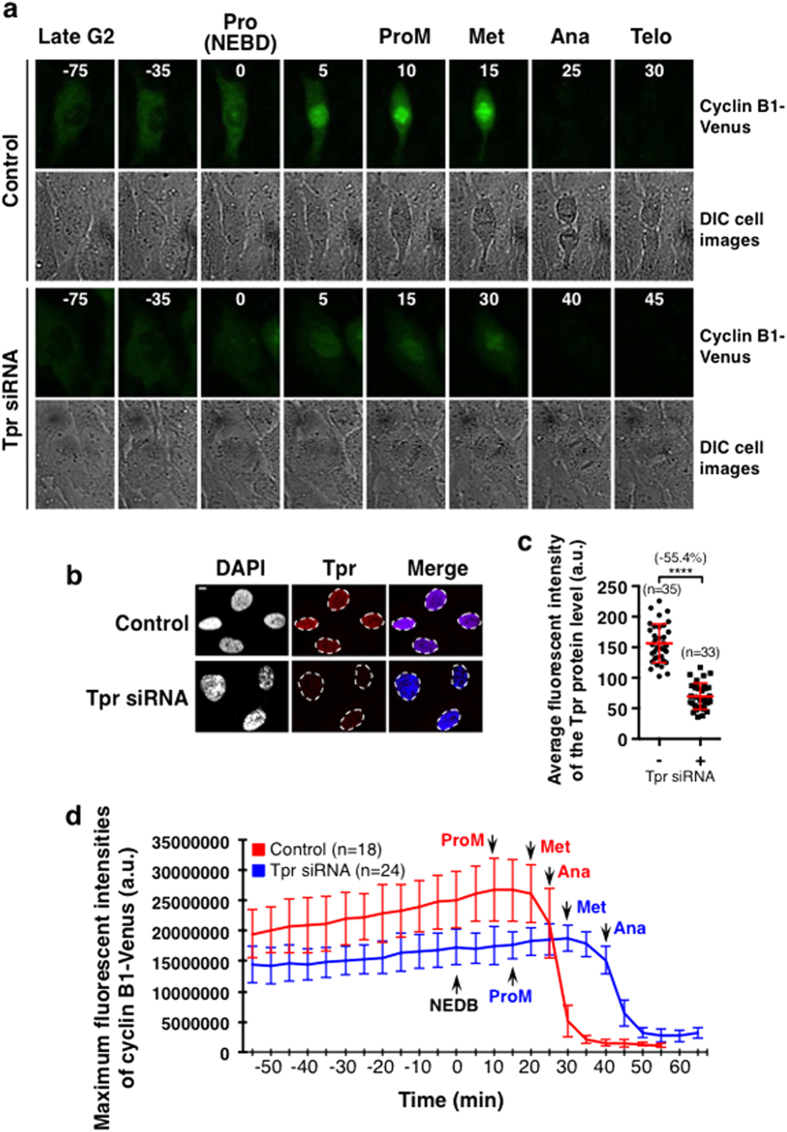
The dynamic accumulation of cyclin B1-venus in RPE1CNNB1-venus cells is significantly reduced after partial depletion of the Tpr proteins using siRNA. (**a**) The maximum projected z-stack confocal time-lapse images showing the fluorescent intensities of the cyclin B1-venus (green top panels) over the time course at selected stages as indicated. DIC images (grey) are also shown together with the relevant cyclin B1-venus images for help in determining the cell cycle stages. RPE1CNNB1-venus cells were treated with or without the Tpr siRNA for 72 hours prior to recording the cyclin B1-Venus for 24 hours with a 5 minute interval using a NIKON A1R fully automated high-speed confocal imaging system at 37 °C and with 5% CO_2_. (**b**) Confocal images comparing the Tpr protein levels in control and Tpr siRNA RPE1CNNB1-venus cells. Tpr (in red) was stained with a rabbit primary antibody to Tpr (sc-67116, Santa Cruz) combined with a rabbit Cy5 secondary antibody (Jackson IRL). DNA was stained with DAPI (in grey and blue in merged images). (**c**) Quantitative results comparing the Tpr protein levels in control and the Tpr siRNA RPE1CNNB1-venus cells measured from nuclear regions (encircled dash lines) of individual cells (in Tpr column in b). (**d**) Quantitative results comparing the average of the maximum fluorescent intensities of the cyclin B1-venus at time intervals as indicated from individual healthy cells between the control (red line, n = 18) and the Tpr siRNA (blue line, n = 24) RPE1CNNB1-venus cells. Specific cell cycle stages in mitosis have been highlighted and the standard deviation error bars were shown. *p < 0.0109; **p < 0.0029; ***p < 0.0008; ****P < 0.0001. n: The number of cells used for quantification. Scale Bar = 5 μM.

Cyclin B1 begins to express and accumulate in the cytoplasm of late G2 and early prophase cells^42^, it has been found that MAD1L1-null cells not only initiated the rapid phase of cyclin B1-Venus degradation after NEBD but also accumulated less cyclin B1-Venus in late G2^41^. Mad1 and Mad2 have been found localised at the nucleoplasmic side of the nuclear membrane and interacted with Tpr to facilitate the interaction between CDC20 and MAD2^37^,^39^,^43^,^44^. Therefore, the PCM complex might function to allow accumulation of cyclin B1 during the cell cycle stages before NEBD. To test this, HeLa cells were stained with a cyclin B1 antibody after Tpr had been knocked out using siRNA, and the results show that the endogenous cyclin B1 levels were significantly lower in prophase cells in comparison to the control (Fig. 5a,b). As Tpr siRNA induced many cell deaths, these indirect antibody fluorescent staining signals are difficult to interpret as some might represent cells that are dying rather than undergoing slippage^40^. To further confirm the premature reduction of cyclin B1 in cells after Tpr was depleted, we analysed the accumulation and destruction profiles of endogenous cyclin B1 in living cells using an RPE1CCNB1-venus knock-in cell line (RPE1: Retina pigment epithelial cell, human), which produces a functional fusion protein^6^. The cyclin-B1–Venus protein accumulated and was destroyed with similar kinetics to the untagged endogenous protein^6^. After 72 hours treatment with or without the Tpr siRNA, the live images of the CCNB1-venus were recorded every 5 minutes for 24 hours using a NIKON A1R fully automated high-speed confocal imaging system. The cell samples were fixed after the recording, immunostained with a Tpr antibody and the reduction in the Tpr proteins quantified. Representative time-lapse images comparing the cyclin B1-venus fluorescence intensities are shown in Fig. 6 (Fig. 6a and S2 MOV1 and 2). Quantitation of the maximum fluorescence signals produced from those individual cells show continuously cycling, indicating that they were not dying (Fig. 6d). Both quantitative and time-lapse imaging results revealed that Tpr siRNA cells not only accumulated significantly less cyclin B1-Venus at metaphase (−44.8%), prometaphase (−42.3%) and prophase at NEBD (−33.3%), but it was also reduced as early as late G2 (Fig. 6d), despite there being only a 55% reduction in the Tpr protein level at the end of recording (Fig. 6b,c). This is consistent with previous observations of cyclin B1-Venus before NEBD when Mad1 was knocked out from the same RPE1 cell line^41^. Consistent with the lower levels of cyclin B1 in these Tpr siRNA cells, the results reveal that the cell cycle was delayed at each mitotic stage (Fig. 6a and d). Bringing all of these results together suggests that this PCM complex is required to allow the accumulation of sufficient cyclin B1 in prophase before NEBD independent of the SAC for maintaining CDK1 kinase activity driving cell to enter mitosis.

### The SAC strength, in terms of CDC20-MAD2 complex formation, cannot be maintained at persistent and constant levels under prolonged nocodazole treatment

**Figure 7:**
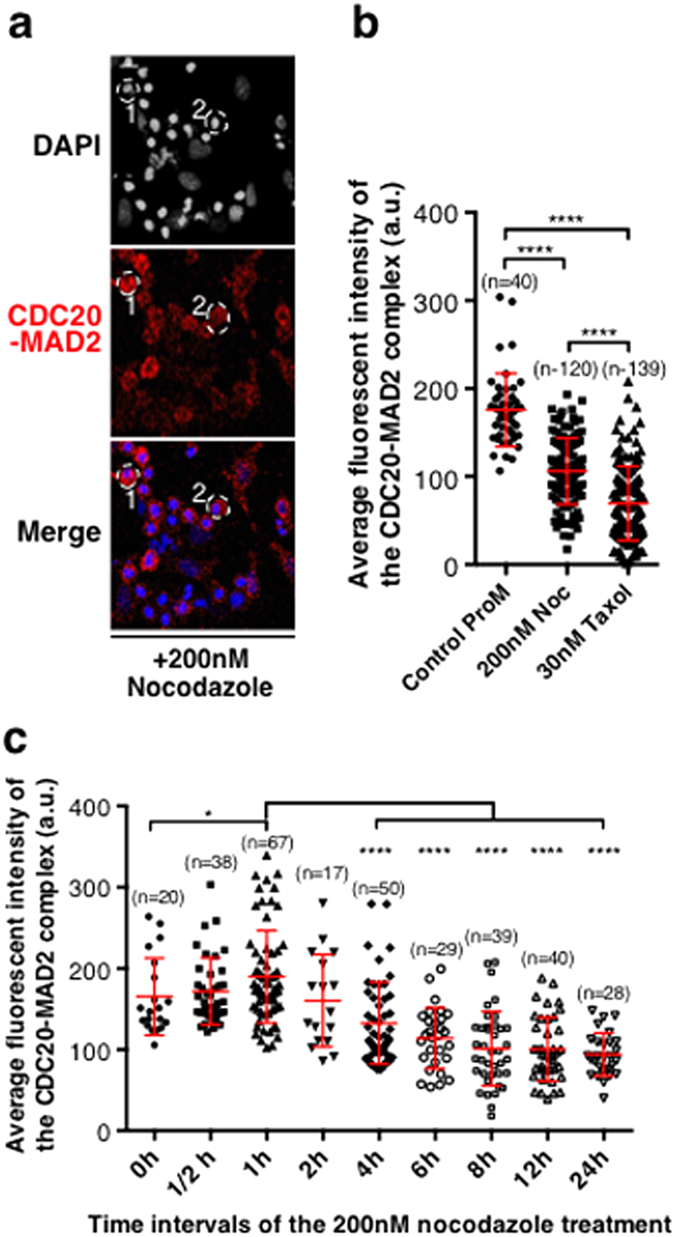
The level of the complex formed between CDC20 and MAD2 in response to nocodazole or taxol treatment at various stages of the cell cycle. (**a**) HeLa cells were treated with 200 nM nocodazole (Noc) or 30 nM taxol for 12 hours and fixed with cold methanol before they were examined using PLA for CDC20 and MAD2 interaction (in red). DNA was stained with DAPI (grey in the top images and blue in the merged images). Scale bar = 50 μM. White dash lines showing #1 & 2 cells: example of nocodazole arrested prometaphase-like cells. (**b**) Comparison of the quantitative results of the average fluorescent intensities of CDC20-MAD2 from individual cells. ProM: Normal prometaphase, Noc: Nocodazole. Cell number used for quantifications: ProM (n = 40), 200 nM Noc ProM (n = 120), 30 nM taxol ProM (n = 139). (**c**) Quantitative results of the average fluorescent intensities of CDC20-MAD2 interaction from individual cells after treated with 200 nM nocodazole at time intervals as indicated. *P < 0.015, ****P < 0.0001. n: The number of cells used for quantification.

The SAC will be robustly activated under anti-mitotic drug treatment, thus its strength (in terms of MCC formation) might be higher compared to that seen in prometaphase, when it is at the highest level under normal mitotic progression (Fig. 3). To test this, HeLa cells were treated for 12 hours with either nocodazole or taxol as described^28^,^45^, and the amounts of the complex formed between CDC20 and MAD2 from individual cells were quantified (Fig. 7a-1) and compared to those found in untreated cells at similar stages (prometaphase) of the cell cycle. The average fluorescence intensity of the interaction between CDC20 and MAD2 varied widely in both treated and untreated prometaphase cells (Fig. 7b) and, surprisingly, the average of the quantitative results showed that the amounts of the CDC20-MAD2 complex detected after 12 hours of treatment were actually significantly lower than those found in cells at prometaphase under normal mitotic progression (Fig. 7b). To test if these low levels of the CDC20-MAD2 complex in nocodazole or taxol treated cells were the consequence of the prolonged mitotic arrest, HeLa cells were fixed at different time intervals after treatment with nocodazole as indicated in Fig. 5c and the levels of the CDC20-MAD2 complex were analysed. The results showed that the overall levels of the CDC20-MAD2 complex increased in response to the nocodazole-provoked SAC activation, and peaked around one hour after the treatment (at about a significant 24.6% increase), the levels then gradually but significantly reduced over time, for instance, by about 47.1% at 12 hours and 50.4% at 24 hours (Fig. 7c). This confirms that the SAC strength, in terms of CDC20-MAD2 complex formation, cannot be maintained at persistent and constant levels under prolonged nocodazole treatment. Additionally, the average fluorescence intensity of the CDC20-MAD2 complex seen in cells treated with 0.03 μM taxol is significantly lower than that seen in 200 nM nocodazole-treated cells (Fig. 7b) which may suggest that a different SAC strength is created in response to different unsatisfied SAC conditions.

### CDC20-MAD2 levels correlate with the levels of cyclin B1 and DNA morphology in nocodazole or taxol treated HeLa cells

**Figure 8:**
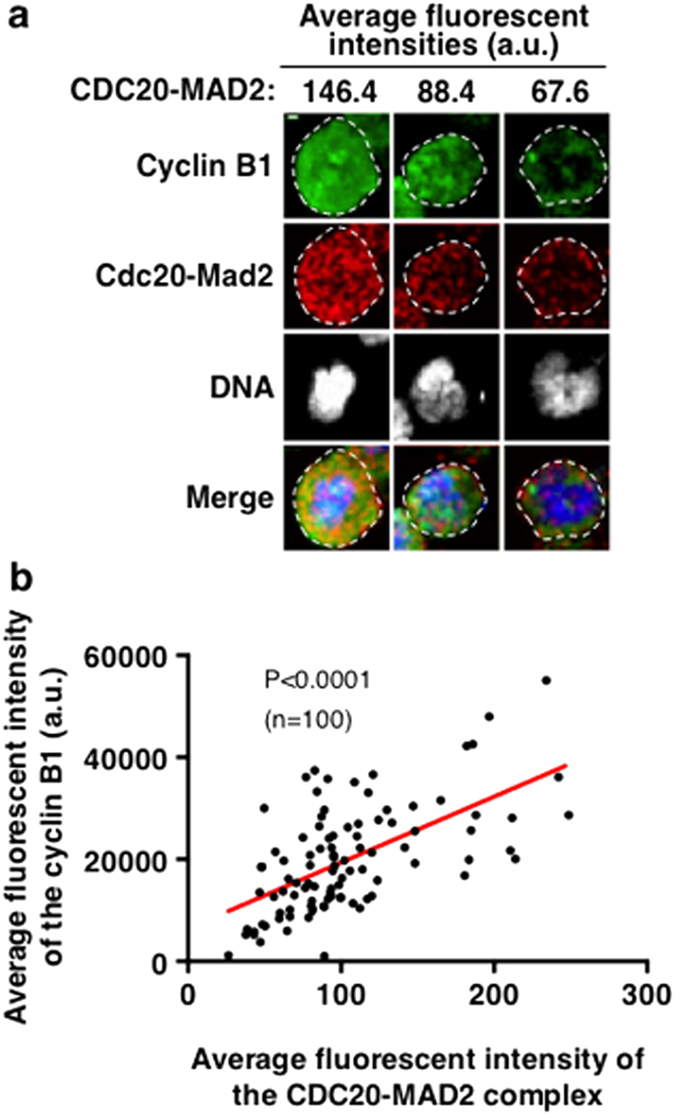
CDC20-MAD2 complex fluorescent signal levels correlate with the levels of cyclin B1 in HeLa cells treated with 200 nM nocodazole. (**a**) Examples of projected confocal images produced from relevant Z-stacks revealing three different cells with correlation between different levels of cyclin B1 (green) and CDC20-MAD2 PLA signals (red). Cyclin B1 protein levels (green) were detected using a rabbit anti-cyclin B1 (Santa Cruz, H-433, sc-752) staining after the completion of the PLA of CDC20-MAD2 (in red). The average fluorescent intensities (units in a.u.) of CDC20-MAD2 from the regions with dashed lines encircling these three cells were shown above the images. The higher level of cyclin B1 correlates with higher numbers of the CDC20-MAD2 complex. DNA was stained with DAPI (in grey or blue in merged images). Scale bar = 5 μM. (**b**) Diagrams showing the correlations between the average fluorescent intensities of cyclin B1 and the average fluorescent PLA signals of CDC20-MAD2 quantified from individual arrested cells after 12 hours treatment with 200 nM nocodazole as described in A. XY frequency distribution from PRISM was used to assess the significance of the correlations between two groups as indicated. The P value was < 0.0001, showing strong correlation. n: The number of cells used for quantification.

It has been suggested that, in mammalian cells exposed to anti-mitotic agents, the APC/C activity cannot be completely inhibited under the persistent active checkpoint signal as cyclin B1 is still slowly degraded and this eventually leads to mitotic exit, a process known as mitotic ‘slippage’^19^,^45^. The rates of slippage differ both within a population and between populations, and correlate with cell fate in many instances^18^. This has been thought to be the cause of the resistance to taxol in clinical cancer therapies^18^. Therefore, it is important to determine the factors that cause the slow degradation of cyclin B1 during prolonged mitotic arrest. Our observation of the unstable (potential auto-disassembly) of the CDC20-MAD2 complex in nocodazole or taxol arrested cells prompted us to determine the relationship between this phenomenon and substrate destruction. HeLa cells were treated for 12 hours with nocodazole and fixed in the same manner as described above. A single cyclin B1 antibody fluorescent staining was applied after the completion of the CDC20-MAD2 PLA staining to avoid any potential interference from each other. Projected confocal images from Z-stacks under identical conditions were used to quantify the average fluorescent intensities of CDC20-MAD2 PLA and cyclin B1 respectively to represent the whole volume of individual arrested cells from the selected regions as indicated (Fig. 8a). The collective data that represents CDC20-MAD2 PLA and cyclin B1 were then plotted against each other using Prism XY frequency distribution (Fig. 8b). The results show that there is a significant correlation between cyclin B1 levels and the levels of the CDC20-MAD2 complex (representing the MCC) that remained in the arrested cells, so the lower the level of the complex the lower the level of cyclin B1 (Fig. 8a and b). Thus, it is likely that the slow decline of the CDC20-MAD2 complexes has weakened the SAC strength, which in turn slowly releases the APC/C activity allowing cyclin B1 destruction.

## Discussion

Biochemical studies have suggested that the MCC, which consists of the CDC20-MAD2 and BUBR1-BUB3 sub-complexes^29^, exists in interphase HeLa cell extracts^10^,^28^,^41^. However, neither the MCC nor its two sub-complexes have ever been visualized in individual cells *in vivo*. Here, for the first time, we have provided the profile of the CDC20-MAD2 complex throughout the cell cycle using a proximity ligation assay of fixed HeLa cells. By examining the CDC20-MAD2 complex in large numbers of individual non-perturbed cells we can confirm that very little or none of this complex exists in interphase cells. The discrepancy between these observations and those previously published is most likely caused by the different methodologies applied, as the biochemical preparation of the interphase extracts would often still contain a substantial population of prophase cells. Additionally, the drugs used for synchronisation of the cell populations may impact on cell cycle organization and the information seen from the cellular compartments in comparison with the non-perturbed cell^46^,^47^,^48^,^49^. This “interphase” form of the MCC studied using biochemical approaches might well be the specific form that we observed in prophase cells as our observation is based on single cell quantification and PLA is one of few methods available for analyzing the interactions between proteins in their native state in individual cells, thus avoiding biochemical extraction or the creation of exogenous over-expressed fusion proteins. PLA can also be used to assign signals to specific subcellular locations such as the cytoplasm or the nucleus^21^, though it might not provide sufficient accuracy to localize the signals to superstructures like the kinetochores.

Our observations reveal that the formation and disassembly of the CDC20-MAD2 complex is controlled in a bell shaped dynamic manner and that the average fluorescence intensity of the complex peaks at prometaphase. This provides new insight into how the SAC strength is controlled, which neither supports the claim that the inhibition of the APC/C is due to the selective activation of the MCC, rather than changes in the amount of the MCC being generated^10^,^28^, nor the popular kinetochore-dependent “on and off” mechanism^1^,^2^. The “rheostat switch” concept for describing the SAC strength was first introduced when the different rates of substrate (cyclin A2) degradation were detected after the SAC was provoked using different concentrations of drugs or by eliminating MCC production either by depleting MAD2, or by inhibiting MPS1^6^. Our findings, however, show the molecular evidence, for the first time, to indicate that the CDC20-MAD2 complex in non-perturbed HeLa cells is regulated to support different strengths of the SAC at different stages of mitosis. Only a basal level of CDC20-MAD2 complex was found in interphase cells (Fig. 3c), this is likely due to low levels of CDC20 presented and it only started to increase after late G2, which is what makes the interaction possible. However, we do confirm that there is an accumulation of CDC20-MAD2 (PCM) complex in prophase cells before NEBD (Fig. 1a). The formation of this PCM requires Tpr, the nuclear pore complex component, which has been previously suggested to facilitate the formation of the CDC20-MAD2 complex in interphase cell extracts^37^,^39^,^41^,^43^,^44^. As Tpr is required for the formation of PCM complex before NEBD, a stage in which the SAC is generally believed to not be activated, but does not affect the formation of the complex in prometaphase and metaphase cells, this suggests that both the kinetochore-dependent and kinetochore-independent pathways are involved in generating inhibitory signals of the APC/C at specific mitotic stages. Whether or not this PCM complex should also be defined as part of the SAC mechanisms still needs to be discussed, as currently it is widely accepted that the SAC “on” signal is generated exclusively by the kinetochores after NEBD^1^,^2^.

Our quantitative results showed that in the presence of nocodazole or taxol the increased level of the CDC20-MAD2 complex will gradually decline over time (Fig. 7c) implying that the amount of the complex formed between CDC20 and MAD2, which reflects the SAC strength, is unstable, and agrees with the suggestion that there is a positive causal relation between MCC levels and the strength of the SAC^6^. Our observations that the reduced levels of CDC20-MAD2 correlate with lower cyclin B levels suggest that an appropriate level of the MCC is required to maintain the SAC strength and that the previous view that the APC/C activity could not be completely inhibited under a persistently active SAC in response to prolonged mitotic arrest was in fact due to a reduction in the SAC strength caused by the slow decline in the level of the CDC20-MAD2 complex.

## Materials and Methods

### Antibodies and reagents

#### Primary antibodies

Rabbit polyclonal anti-CDC20 antibody (Abcam, ab26483), mouse monoclonal anti-p55 CDC (E-7) (Santa Cruz Biotech, sc-13162), rabbit anti-MAD2 (Bethyl, A310-082A, which has been recommended for PLA use), mouse monoclonal anti-cyclin B1 (BD Biosciences, material No. 554178), mouse monoclonal anti-cyclin B1 (GNS) (Santa Cruz, sc-245), rabbit polyclonal anti-cyclin B1 (H-433) antibody (Santa Cruz, sc-752), (mouse monoclonal anti-tubulin DM1α + β (Abcam, ab44928), rabbit anti-pericentrin antibody (Abcam, ab4448), Goat anti-Mouse IgG (H + L) (62–6500) and Goat Anti-Rabbit IgG (H&L) (A24533), unconjugated and affinity purified antibodies (Life technologies).

#### Secondary Antibodies

goat polyclonal secondary antibody to mouse IgG- H + L (FITC) (Abcam, ab6785), goat-anti human IgG (H + L) fluorescein conjugated (Thermo Scientific, product number: 31529). (Invitrogen), IRDye 680 donkey anti–mouse (926–322227; LI-COR Biosciences), and IRDye 800CW donkey anti–mouse (926–32212; LI-COR Biosciences).

#### Duolink reagents

Duolink *In Situ* PLA probe anti-Rabbit PLUS, Duolink *In Situ* PLA probe anti-Mouse Minus, Duolink *In Situ* Detection Reagents Red (Duolink assay reagent kits was distributed by Sigma-Aldrich).

#### Chemicals

M2I-1 (ChemBridge Corporation), CelLytic^™^ MT Cell Lysis Reagent (Sigma-Aldrich, C3228), Protease inhibitor cocktail (Sigma-Aldrich, p8340), Reversine (C656820-32-5), MG132 (Cayman Chemical, 133407-82-6).

### Cell culture conditions and treatments

HeLa cells or the RPE1CCNB1-Venus cells (a gift from Jonathan Pines’ lab) were cultured in DMEM (Sigma) medium along with 10% calf serum, glucose, non-essential amino acids and penicillin streptomycin in a 37 °C incubator with 5% CO_2_ with routine medium changes. After being split, 1 ml DMEM that contained 20,000 HeLa cells was seeded onto 10 mm diameter coverslips (prior washed with 100% ethanol), which were placed in the wells of a 24 well culture plate. The plate was then kept in an incubator with 5% CO_2_ at 37 °C until 70% confluence was reached. Cells were treated for 16 hours with 30 nM Taxol^45^ and 200 nM nocodazole^28^ or with 50 μM M2I-1 (in 0.5% DMSO)^33^ respectively for immunofluorescent staining and western blotting. Subsequently the treated or untreated normal HeLa cells on the coverslips were fixed using 1 ml cold (−20 °C) methanol, left at room temperature for 5 minutes and then stored at −20 °C for use.

For the MPS1 inhibition with reversine, HeLa cells were released after 7 hours from double thymidine synchronization and treated with either DMSO as the control or 0.5 μM reversine in DMSO for 30 minutes, followed by 3 hours in a solution containing either 10 μM MG132 alone or one containing 10 μM MG132 + 0.5 μM reversine to halt the cells in mitosis and to prevent CDC20 degradation^35^. After the treatment, cells on the coverslips were fixed and kept as described above.

Cells fixed with 4% paraformaldehyde would normally retain better DNA morphologies under the microscope, but we used the cold methanol throughout the project, because cyclin B1 and CDC20 are rapid turnover proteins and so detecting their protein levels and distribution is crucially dependent on fixation conditions and the cold methanol fixation has been proved to be the appropriate method for this^50^.

### Immunoblotting

The appropriate cells were collected by centrifugation at 150 g for 5 minutes after trypsinization and washed once with pre-warmed PBS. Cells then were lysed in an appropriate amount of CelLytic^™^ MT Cell Lysis Reagent (Sigma-Aldrich, C3228) containing 1x protease inhibitor cocktail (Sigma-Aldrich, p8340) on ice with agitation for 30 minutes. Cell lysates were added to Laemmli sample buffer and boiled for 5 min. Samples were separated on a precast 10% Bis-Tris SDS-PAGE gel. Proteins were transferred to nitrocellulose membranes and these were blocked in 1x Odyssey blocking solution for 1 hour. The membranes were probed with appropriate primary antibodies in Odyssey blocking solution (1:500 dilution) at room temperature for 2 hours or at 4 °C overnight with agitation. The membrane was then incubated with a secondary antibody solution of IRDye 680 donkey anti–mouse, or IRDye 800CW donkey anti–mouse (from Invitrogen), at 1:10,000 dilution where appropriate for one and a half hours. The fluorescence signals were detected using a CCD scanner (Odyssey; LI-COR Biosciences) according to the manufacturer’s instructions.

### PLA and conventional fluorescent immunostaining

#### PLA

The fixed HeLa cells on the coverslip were rehydrated with 1 × PBS and probed with the chosen pairs of primary antibodies according to the standard commercial protocol modified as follows. The coverslips were blocked with the PLA blocking solution for 10 minutes prior to the application of 20 μl of the primary antibody at a 1:250 dilution in the PLA antibody diluent. The coverslip was kept in a wet chamber and incubated for 120 minutes at 37 °C. Subsequently the coverslips were washed 3 times with 1 × PBS for 5 minutes with gentle agitation. 15 μl of the appropriate PLA secondary probe at a 1:5 dilution were then added onto each coverslip and they were incubated at 37 °C for 60 minutes followed by washing twice with buffer A for 5 minutes with gentle agitation. 15 μl of the ligation mix was then applied to each of the coverslips to complete the ligation process at 37 °C for 30 minutes followed by washing twice with buffer A for 2 minutes with gentle agitation. These coverslips were then incubated with 15μl polymerisation mix and incubated at 37 °C for 120 minutes. Following the incubation, the coverslips were washed twice with buffer B for 2 minutes with gentle agitation. This was followed by one further wash with buffer B for 10 minutes with no agitation. After another 2 minutes, the coverslips were washed with 0.01% buffer B three times with no agitation.

#### Conventional fluorescent immunostaining

After the completion of the PLA staining, the cells on the coverslips were then probed with either 20 μL mouse monoclonal Dm1α + β antibodies, or anti-pericentrin antibody or anti-cyclin B1 antibody (1:500 in PLA diluent) for labelling the microtubules, centrosomes or cyclin B1 respectively, and incubated overnight at 4 °C. This was followed by incubation with the goat polyclonal secondary antibody to FITC conjugated mouse IgG (H + L) for probing with the DM1α + β primary antibodies or goat anti-rabbit IgG (H + L) (DyLight 488) for probing with pericentrin antibody (all at 1:500 dilutions in PLA diluent). The secondary antibody solution also contains DAPI (1:3000) for DNA staining. This was incubated at room temperature for 1 hour. Finally, the coverslips were washed three times for 5 minutes with 1 × PBS. The coverslips were mounted with mounting solution after they were air-dried.

### RNA interference

The Dharmacon siGENOME human TPR (7175) siRNA-SMARTpool (M-010548-02-005), ON-TARGETplus positive control of GAPDH (D-001830-02) and non-targeting control (D-001810-01) oligonucleotides were used for knockout of the Tpr proteins from HeLa cells. Cells were transfected with 40 nM of oligonucleotide using DharmaFECT 1 transfection reagent (T-2001-01). Cells were re-transfected with the same amount of the oligonucleotide at 48 hours after first transfection. Cells were collected at 72 or 96 hours after the second transfection for conventional immunofluorescent staining, confocal imaging or Duolink based proximity ligation assay using two primary antibodies, or cells were collected for western blot analysis.

### Confocal imaging and quantification of the fluorescent complexes

The stained HeLa cells on the coverslips were scanned using a Leica TCS SP2 laser scanning confocal system with a 40x oil objective lens and using the following excitation wavelengths: 405 nm for detecting DAPI (DNA), 488 nm for FITC (detecting the microtubules) and 594 nm for detecting the TexasRed (interaction complex between the protein pairs of interest). The cell cycle stages of interest were determined using the DNA and microtubule morphologies (stained with DM1α + β antibodies) or centrosomes (stained with pericentrin antibody). Sequential scanning was used to avoid interference between channels. The Z-stack section scanning with a consistent distance of 0.3 μM between sections was used to cover and collect the whole volume of the fluorescent intensity information for calculation and comparison. These Z-stack section images were projected to produce a single image for quantification of the collective maximum fluorescent intensity to represent the whole volume of the cell or in selected regions of interest (Fig. S1).

The live images of the CCNB1-venus IN RPE1 CELLS were recorded using a NIKON A1R fully automated high-speed confocal imaging system with a time interval of 5 minutes over 24 hours.

ImageJ, Photoshop, IMARIS and NIS-elements were used for quantification, editing of the fluorescent intensities of the complex or live imaging processing where appropriate.

## Additional Information

**How to cite this article**: Li, J. *et al*. The kinetochore-dependent and -independent formation of the CDC20-MAD2 complex and its functions in HeLa cells. *Sci. Rep.* **7**, 41072; doi: 10.1038/srep41072 (2017).

**Publisher's note:** Springer Nature remains neutral with regard to jurisdictional claims in published maps and institutional affiliations.

## Supplementary Material

Supplementary Information

Supplementary movie S1

Supplementary movie S2
