# Supplementary material for: The kinetochore-dependent and -independent formation of the CDC20-MAD2 complex and its functions in HeLa cells
Source: Sci Rep. 2017 Jan 23;7:41072. doi: 10.1038/srep41072 (PMC5253641; doi:10.1038/srep41072)
Supplement: Supplementary Information [file srep41072-s1.pdf]

The kinetochore-dependent and -independent formation of the CDC20-MAD2 complex and its functions in HeLa cells

Jianquan Li[1*], Nanmao Dang[1*], Daniel James Wood[1,2] and Jun-Yong Huang[1,3]
[1]Institute for Cell and Molecular Biosciences, Newcastle University, Framlington Place, Newcastle upon Tyne, NE2 4HH, UK. [2]Current address: Northern Institute for Cancer Research, Paul O'Gorman Building, Newcastle University, Framlington Place, Newcastle upon Tyne, NE2 4HH, UK. *: These authors have made an equal contribution. [3]Correspondence should be addressed to J-Y.H.
(Email: junyong.huang@newcastle.ac.uk).

Supplementary figures:

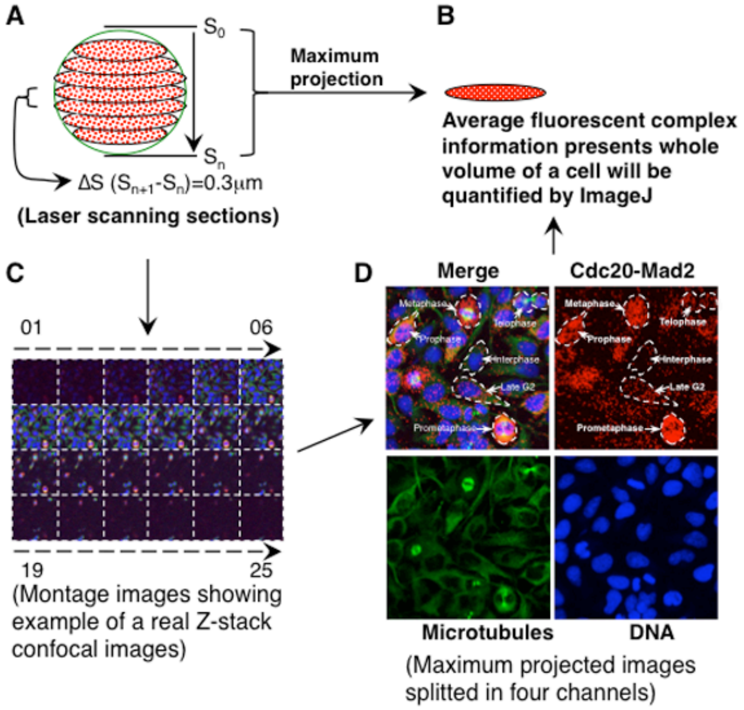

Figure S1. Confocal scanning and ImageJ quantification of CDC20-MAD2 PLA signals from individual HeLa cells. (a). The diagram illustrates sections of a Z-stack representing a cell with a 0.3μm interval between sectional images applied consistently to all the cells throughout this project. (**b**). A diagram showing the projected images from the Z-stack sections, which would be used for quantification of the average PLA

fluorescent intensities across the entire cellular area using ImageJ. **(c).** An example of a real montage of confocal images from a Z-stack of HeLa cells with CDC20-MAD2 complex PLA signals (in red), microtubules (DM1α antibody staining in green) and DNA (DAPI staining in blue). Dashed-line arrows indicate the order of the section images from 01 to 25. **(d).** Maximum projected confocal images produced from the Z-stack in (C), showing the CDC20-MAD2 PLA information from individual cells at different cell cycle stages as highlighted in the field of view. The individual image channels were split to display the CDC20-MAD2 PLA information in red, DNA (DAPI staining) in blue and the microtubules in green. DNA and microtubule morphologies were used to determine the cell cycle stages. Some specific cell cycle stages have been highlighted in the merged and CDC20-MAD2 PLA images. The white dashed-line square boxes were used to separate the individual sections in the montage images. The PLA signals representing the interaction between CDC20 and MAD2 were produced using mouse monoclonal anti-CDC20 antibody (Santa Cruz, p55CDC (E-7, sc-13162) and rabbit anti-MAD2 (Bethyl, A310-082A).

Mov. S1. A time-lapse movie showing the cyclin B1-venus fusion protein expression profile (in green in the left hand panel) and the relevant DIC (in grey in the right hand panel) in an RPE1CNNB1-venus cell treated with a negative control siRNA for Tpr for 72 hours prior to imaging. Digital time intervals are shown on the lower left in white. The 00:00 time point was used to indicate the starting point of NEBD. The RPE1CNNB1-venus cells were imaged for 24 hours at 5 minute intervals using a NIKON A1R fully automated high-speed confocal imaging system at 37°C and provided with 5% CO_2 incubating conditions.

Mov. S2: A time-lapse movie showing the cyclin B1-venus fusion protein expression profile (in green in the left hand panel) and the relevant DIC (in grey in the right hand panel) in an RPE1CNNB1-venus cell treated with Tpr siRNA for 72 hours prior to imaging. Digital time intervals are shown on the lower left in white. The 00:00 time point was used to indicate the starting point of NEBD. The RPE1CNNB1-venus cells were imaged for 24 hours at 5 minute intervals using a NIKON A1R fully automated high-speed confocal imaging system with the conditions identical to those described in MOV. S1.
